# Fertility in patients with Hirschsprung's disease: population-based cohort study

**DOI:** 10.1093/bjsopen/zrad043

**Published:** 2023-06-09

**Authors:** Cornelia Byström, Lisa Örtqvist, Anna Gunnarsdóttir, Tomas Wester, Anna Löf Granström

**Affiliations:** Department of Women’s and Children’s Health, Karolinska Institutet, Stockholm, Sweden; Department of Women’s and Children’s Health, Karolinska Institutet, Stockholm, Sweden; Unit of Paediatric Surgery, Karolinska University Hospital, Stockholm, Sweden; Department of Women’s and Children’s Health, Karolinska Institutet, Stockholm, Sweden; Unit of Paediatric Surgery, Karolinska University Hospital, Stockholm, Sweden; Department of Women’s and Children’s Health, Karolinska Institutet, Stockholm, Sweden; Unit of Paediatric Surgery, Karolinska University Hospital, Stockholm, Sweden; Department of Women’s and Children’s Health, Karolinska Institutet, Stockholm, Sweden; Unit of Paediatric Surgery, Karolinska University Hospital, Stockholm, Sweden

## Abstract

**Background:**

The aim of this study was to assess fertility in patients treated for Hirschsprung’s disease.

**Methods:**

This was a nationwide, population-based cohort study, including all patients with Hirschsprung’s disease registered in the Swedish National Patient Register between 1964 and 2004. Five age- and sex-matched controls per patient were randomly selected by Statistics Sweden. Outcome data were retrieved from the Multi-Generation Register, and the Swedish National Patient Register. Study exposure was Hirschsprung’s disease and the primary outcome was fertility, defined as having one or more children. Individuals with chromosomal anomalies were excluded.

**Results:**

The study cohort comprised 597 patients with Hirschsprung’s disease (143 female) and 2969 controls (714 female). The mean(s.d.) age at follow-up was 29.6(10.0) years for patients and 29.8(10.1) years for the controls. A total of 191 (32.0 per cent) patients compared with 1072 (36.1 per cent) controls had one or more children (*P* = 0.061). The analysis showed that fewer female patients with Hirschsprung’s disease had a child (29.4 *versus* 38.7 per cent, *P* = 0.037), they were older when they gave birth to their first child (28.1 *versus* 26.4 years, *P* = 0.033), and they had fewer children. Of the female patients with Hirschsprung’s disease, 19 (45.2 per cent) had only one child, compared with 79 (28.6 per cent) of the female control group (*P* = 0.047). No difference was noted in the male group in this regard.

**Conclusion:**

Female patients with Hirschsprung’s disease were less likely to have a child, had fewer children, and were older when they gave birth to their first child compared with the controls, indicating impaired fertility. There was no significant difference between male patients with Hirschsprung's disease and controls.

## Teaser

The aim of this nationwide, register-based cohort study, including all patients with Hirschsprung’s disease registered in the Swedish National Patient Register between 1964 and 2004, and five age- and sex-matched controls per patient, was to assess fertility in patients treated for Hirschsprung’s disease. The results showed that females with Hirschsprung’s disease were less likely to be mothers, had fewer children, and were older when they gave birth to their first child compared with the controls, indicating impaired fertility.

## Introduction

Hirschsprung’s disease (HSCR) is a congenital bowel disorder characterized by aganglionosis along variable lengths of the distal hindgut, leading to obstruction due to reduced bowel motility^1–3^. Treatment comprises removal of the aganglionic colon and anastomosis of the normally innervated proximal bowel to the anorectum in early life. The most commonly performed operations used to be the Swenson, Duhamel, and Soave procedures^2^. Since the 1990s, transabdominal two- or three-stage procedures have largely been replaced by minimal-access, one-stage surgery, such as laparoscopic-assisted pull-through and transanal pull-through^1,2^. Potential short-term complications include anastomotic leakage and stricture, postoperative Hirschsprung-associated enterocolitis, bleeding, perianal excoriation, and wound infection^1,4–6^. Adhesive small bowel obstruction has been described as occurring in 5 per cent of patients with HSCR after minimally invasive surgery and in up to 29 per cent of patients after laparotomy^6,7^. Impaired bowel function, both incontinence and constipation, is the most common long-term complication^1,4,8^.

The surgical procedure for HSCR includes dissection in the pelvis, close to the fallopian tubes and ovaries in female patients and to the seminal vesicles and vas deferens in male patients. The surgical area is also close to the inferior hypogastric plexus and its branches, and some of the surgical methods used for HSCR include dissection of the anterior aspect of the rectum. Reduced fertility in female patients has previously been noted after other pelvic surgical operations, such as ileal pouch anal anastomosis for ulcerative colitis or familial adenomatous polyposis^9,10^. This is believed to be partially due to postoperative adhesions disturbing fallopian tube function or nerve injury. Fertility data in male patients who have undergone the same procedures are conflicting^11,12^.

Very few studies, all limited by a small sample size, have addressed fertility and sexual function in patients with HSCR. Sexual dysfunction has been suggested in both men and women^13,14^. A decreased chance of conceiving has also been suggested in female patients with HSCR who have tried to become pregnant^14^. A correlation between bilateral hydrosalpinx and HSCR has also been suggested^15,16^.

Currently, no studies have been conducted on fertility in individuals with HSCR compared with controls from the general population. The aim of this study was to determine whether individuals with HSCR have impaired fertility.

## Methods

### Study design

This was a nationwide, population-based cohort study, with patients included from 1 January 1964 to 31 December 2017. The study exposure was HSCR diagnosis and the primary outcome was fertility, using the rate of childbirth as a proxy. Secondary outcomes were parental age when the first child was born and number of children. Exposure and outcome were assessed through linkage between the registers held by the Swedish National Board of Health and Welfare, and Statistics Sweden. The STROBE guidelines for cohort studies were followed (see the checklist in the *Supplementary material*)^17^.

### Data resources/registers

All residents in Sweden receive a unique personal identification number at birth or immigration through which linkage between national registers is possible.

The Swedish National Patient Register, established in 1964 by the Swedish National Board of Health and Welfare, contains prospective information regarding inpatients and achieved national coverage in 1987. More than 99 per cent of all Swedish hospital discharges are registered in the Swedish National Patient Register. The data include sex, age, geographical data, diagnoses, and surgical procedures, as well as dates of admission and discharge. Diagnoses are registered according to the ICD. This classification has been modified over the years: ICD-7 from 1964 to 1967, ICD-8 from 1969 to 1986, ICD-9 from 1987 to 1996, and ICD-10 since 1997. The registered diagnoses have been shown to have a validity of 85–95 per cent in the most recent validation of the Swedish National Patient Register in 2011^18^. Outpatient specialist care was included in the register from 2001, but primary healthcare is not covered by the Swedish National Patient Register.

The Multi-Generation Register is a register held by Statistics Sweden that provides information about the linkage between a so-called index person (a person born in 1932 or later, who was registered in Sweden sometime after 1961) and their parents. The register was established in 2000 and the data are from the National Tax Board, including data on more than 9 million individuals. The data include sex, country of birth, date of birth, and information about biological and adopted children. The coverage is almost complete regarding index persons for individuals registered in Sweden since 1961 and data on parents are available for 95–97 per cent of the index persons^19,20^.

The Swedish Register of Education was established in 1985 and records the highest educational level of inhabitants between 16 and 74 years of age. The register is managed by Statistics Sweden and is updated annually^21^.

### Participants

The study cohort was collected from the Swedish National Patient Register. The inclusion criteria were HSCR (ICD-7, 756.31; ICD-8, 751.39; ICD-9, 751D; and ICD-10, Q431) between 1964 and 2004. To reduce the risk of including individuals who had been incorrectly diagnosed with HSCR, only individuals with HSCR as the main diagnosis registered at a paediatric surgical unit were included in the study. Exclusion criteria were death before the age of 13 years, as this corresponded to the minimum age of the participants at the point of data retrieval, and chromosomal anomalies (ICD-10, Q90–99; ICD-9, 758; and ICD-8, 759.30–759.59), as they may affect fertility. For each HSCR patient, five age- and sex-matched controls, randomly selected by Statistics Sweden, were included. The same exclusion criteria were applied to the controls after matching. Controls paired with the excluded HSCR patients were also excluded from the cohort.

### Variables

Data on age and sex were retrieved from the Swedish National Patient Register. Demographic data on marital status, highest educational level, and mortality were collected from Statistics Sweden. Data on highest educational level were categorized into three levels of education: less than or equal to 9 years of compulsory school; two to three years of upper secondary school; and university education. Congenital malformations and chromosomal anomalies comprised the following diagnoses in the Swedish National Patient Register: ICD-8, 740–759.99; ICD-9, 740–759×; and ICD-10, Q00–Q99. The following diagnoses were excluded from congenital malformations in this study: Meckel’s diverticulum (751, 751A, Q430), undescended testis (752.1, 752F, Q531–532, Q539), and urachus remnant (753H, Q644), due to the potential for diagnostic bias.

Fertility was measured as the rate of childbirth, based on data from the Multi-Generation Register. Additionally, the number of children per participant and parental age at birth of first child were also collected from the Multi-Generation Register. Faecal incontinence (ICD-8, 306.70, 785; ICD-9, 787G; and ICD-10, R15.9) and urinary incontinence (ICD-9, 625G, 788D, V53G; and ICD-10, N39, R32) were considered potential confounders, as the stigma surrounding incontinence might cause social or psychological barriers, contributing to fewer intimate relationships and sexual contact. Thus, data on incontinence were collected from the Swedish National Patient Register. Individuals diagnosed with infertility (ICD-8, 606, 628; ICD-9, 606, 628; and ICD-10, N46, N90) were also identified in the Swedish National Patient Register.

### Statistical analysis

Categorical data are presented as frequencies or proportions. Ordinal data were analysed using the Mann–Whitney *U* test, and nominal data were analysed using the chi-squared test (greater than five values per cell)/two-tailed Fisher’s exact test (five or less than five values per cell). Numerical data are presented as mean(s.d.). The numerical data were analysed using a *t* test (normally distributed data) and the Mann–Whitney *U* test (non-normally distributed data). Matched Cox regression was used for adjusted calculations. *P* < 0.050 was considered statistically significant. The association between exposed and unexposed individuals was analysed using the R program^22^.

### Ethics

The study was approved by the Regional Ethics Review Board in Stockholm (2018/2514–31).

## Results

### Participants

**Fig. 1:**
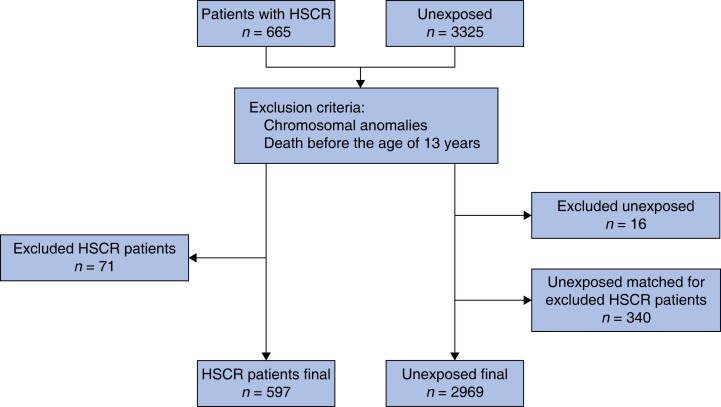
Flow chart showing the inclusion process HSCR, Hirschsprung’s disease.

The final study population comprised 597 HSCR patients and 2969 controls (*Fig. 1*).

### Demographic data

The male : female ratio was 76 : 24 per cent. The mean age at follow-up was 30 years. There was no significant difference in education level between the HSCR patients and controls. Marital status and age at marriage did not differ significantly when comparing the HSCR patients and controls. Female patients with HSCR married later in life compared with female patients from the control group. The prevalence of other malformations (27.3 *versus* 7.8 per cent) and mortality (2.3 *versus* 0.8 per cent) during the observation interval was higher in HSCR patients compared with the controls (*Table 1*).

**Table 1:** Demographic data of the study population

	HSCR (*n* = 597)	Control (*n* = 2969)	*P*
**Sex**			1.000
Female	143 (24.0)	714 (24.0)	
Male	454 (76.0)	2255 (76.0)	
Age at follow-up (years), mean(s.d.)	29.6(10.0)	29.8(10.1)	0.609
**Education level**			
<16 years old	32 (5.7)	162 (5.6)	0.720
≤9 years of compulsory school	101 (8.0)	468 (16.1)	
Upper secondary school	248 (44.2)	1318 (45.3)	
University education	180 (32.1)	959 (33.0)	
NA	36 (6.0)	62 (2.1)	
**Marital status**			
Married	106 (17.9)	544 (18.5)	0.183
Unmarried	469 (79.2)	2314 (78.5)	
Divorced	16 (2.5)	88 (3.0)	
Widow/widower	1 (0.2)	1 (0.0)	
NA	5 (0.8)	22 (0.7)	
Age at marriage (years), mean(s.d.)	30.6(5.1)	29.8(4.7)	0.102
Other malformations	163 (27.3)	233 (7.8)	<0.001*
Mortality during the observation interval	14 (2.3)	25 (0.8)	0.004*

Values are *n* (%) unless otherwise stated. *Statistical significance. HSCR, Hirschsprung’s disease; NA, not applicable.

When men and women were analysed separately, malformations were more common in HSCR patients (27.8 *versus* 8.1 per cent for men and 25.9 *versus* 7.1 per cent for women, *P* > 0.001). Mortality was significantly higher in male HSCR patients during the observation interval (2.6 *versus* 0.9 per cent, *P* = 0.007), but not in female patients (1.4 *versus* 0.6 per cent, *P* = 0.263).

### Fertility data

Out of 597 HSCR patients, 191 (32.0 per cent) had biological children compared with 1072 (36.1 per cent) in the control group (*P* = 0.061). There was no significant difference in the number of children per individual or age at first child between the two groups (*Table 2*).

**Table 2:** Comparison of fertility between HSCR patients and controls

	HSCR (*n* = 597)	Control (*n* = 2969)	*P*
Individuals with a child/children	191 (32.0)	1072 (36.1)	0.061
**No. of children**			
One	71 (37.2)	321 (29.9)	0.051
Two or more	120 (62.8)	751 (70.1)	
Age at birth of first child (years), mean(s.d.)	28.4(4.8)	28.1(4.9)	0.569

Values are *n* (%) unless otherwise stated. HSCR, Hirschsprung’s disease.

Analysing by sex, fewer female HSCR patients had children (42 female HSCR patients, 29.4 per cent) compared with the female controls (276 female controls, 38.7 per cent) (*P* = 0.037). Female HSCR patients with children also had significantly fewer children and gave birth to their first child significantly later in life compared with the control group (*Table 3*). There was no difference in this regard for the male groups.

**Table 3:** Sex comparison of fertility between HSCR patients and controls

	Male patients with HSCR (*n* = 454)	Male controls (*n* = 2255)	*P*	Female patients with HSCR (*n* = 143)	Female controls (*n* = 714)	*P*
**Marital status**						
Married	81 (18.0)	410 (18.3)	0.271	25 (17.6)	134 (18.8)	0.531
Unmarried	357 (79.3)	1757 (78.6)		112 (78.9)	557 (78.3)	
Divorced	11 (2.4)	69 (3.1)		4 (2.8)	19 (2.7)	
Widow/widower	1 (0.2)	0		1 (0.7)	1 (0.1)	
Age at marriage (years), mean(s.d.)	30.4(4.9)	30.2(4.6)	0.836	31.4(5.6)	28.6(4.6)	0.006*
Individuals with a child/children	149 (32.8)	796 (35.3)	0.331	42 (29.4)	276 (38.7)	0.037*
**No. of children**						
One	52 (34.9)	242 (30.4)	0.289	19 (45.2)	79 (28.6)	0.047*
Two or more	97 (65.1)	554 (69.6)		23 (54.8)	197 (71.4)	
Age at birth of first child (years), mean(s.d.)	28.4(4.6)	28.7(4.9)	0.454	28.1(5.5)	26.4(4.7)	0.033*

Values are *n* (%) unless otherwise stated. *Statistical significance. HSCR, Hirschsprung’s disease.

The lower possibility of having children over time for female HSCR patients remained significant after a matched Cox regression analysis adjusted for educational level and malformations other than HSCR (HR 0.73 (c.i. 0.55 to 0.97)). There was no significant difference for male HSCR patients after adjusted analyses (HR 1.06 (c.i. 0.87 to 1.29)).

### Infertility and incontinence

There was no difference in the prevalence of an infertility diagnosis between HSCR patients and controls (2.7 *versus* 2.1 per cent respectively, *P* = 0.358). The prevalence of both faecal and urinary incontinence was significantly higher in HSCR patients compared with the controls (*Table 4*).

**Table 4:** Comparison of prevalence of infertility diagnosis and incontinence

	HSCR (*n* = 597)	Control(*n* = 2969)	*P*
Infertility	16 (2.7)	62 (2.1)	0.358
Urinary incontinence	36 (6.0)	81 (2.7)	<0.001*
Faecal incontinence	74 (12.4)	36 (1.2)	<0.001*
Age at first urinary incontinence diagnosis (years), mean(s.d.)	15.9(12.34)	19.5(11.41)	0.128
Age at first faecal incontinence diagnosis (years), mean(s.d.)	7.8(6.97)	7.9(5.47)	0.912

Values are *n* (%) unless otherwise stated. *Statistical significance. HSCR, Hirschsprung’s disease.

When sex was analysed separately, there was a significantly higher prevalence of faecal incontinence in both female and male HSCR patients (10.5 *versus* 0.7 per cent for women and 13.0 *versus* 1.4 per cent for men, *P* < 0.001). A significant difference in urinary incontinence was only present in male HSCR patients *versus* controls (4.8 *versus* 1.5 per cent respectively, *P* < 0.001) (9.8 *versus* 6.6 per cent respectively for female HSCR patients, *P* = 0.210).

## Discussion

This nationwide, population-based cohort study, using childbirth as a proxy, shows significantly reduced fertility in female HSCR patients compared with population-based controls. Female HSCR patients were also older than the controls when they gave birth to their first child. The same differences did not appear between male HSCR patients and controls. However, the prevalence of an infertility diagnosis did not differ between the patients and controls. Both faecal and urinary incontinence were more common in HSCR patients than in the controls.

Few studies have addressed fertility in HSCR patients, and no previous studies have compared fertility between HSCR patients and population-based controls. Moore *et al*.^23^ suggested that sexual dysfunction, including infertility, was present in 1–10 per cent of patients treated with the Swenson, Duhamel, and Soave procedures. Neither the exact proportion of infertility nor the differences between sexes were specified. In a review by Swenson *et al*.^24^, 80 out of 101 post-pubertal men who had undergone a Swenson’s procedure were married and had a total of 146 children, indicating that their fertility was unaffected, which aligns with the results for the male patients in the current study.

As HSCR is less common in women, there are less data on fertility in women. Similar to the current results, Davidson *et al*.^14^ recently reported the negative impact of HSCR in women. They demonstrated that only 47 per cent of women with HSCR, who had attempted to conceive a child, conceived naturally, compared with 83 per cent of men. They also reported a high prevalence of dyspareunia (50 per cent) in sexually active female HSCR patients. An association has also been suggested between bilateral hydrosalpinx, and reduced fertility, and HSCR^15,16,25^. The association is assumed to be due to surgical complications, such as intra-abdominal adhesions or local damage to nerves that innervate the fallopian tubes. However, pre-existing pathological conditions associated with HSCR cannot be ruled out as the cause of hydrosalpinx^16^. The prevalence of hydrosalpinx was not assessed in this study, but it is possible that it might contribute to the lower rate of childbirth in female HSCR patients. Female HSCR patients have a relatively higher incidence of long-segment disease and total colonic aganglionosis, with more severe symptoms, which might contribute to the difference in fertility only being present between female patients and controls in this study^26,27^.

Ileal pouch anal anastomosis for ulcerative colitis and familial adenomatous polyposis are similar to the surgical treatment for HSCR, and both patient groups often suffer from inflammation of the bowel. The results of an infertility meta-analysis by Waljee *et al*.^10^ were comparable to results from this study, showing reduced fertility in females who had undergone ileal pouch anal anastomosis for ulcerative colitis, while it was reported that male fertility was not affected by ileal pouch anastomosis surgery^11^. Both adhesions and scarring of the fallopian tube were suggested as possible explanations of reduced fertility^10^.

In addition to mechanical damage from surgery and/or inflammation, psychological factors might contribute to the lower rate of childbirth in female HSCR patients. Faecal incontinence was more common in HSCR patients compared with the controls for both women and men. This could potentially have a negative impact on sexual relationships, with fear of soiling as a contributory factor. The different results for male and female HSCR patients could be attributed to different social expectations, better coping mechanisms in male patients with incontinence, and/or different physical demands for men and women during sexual activity. A recent review of faecal incontinence and sexual function in women showed a more negative experience of sexual activity, but no difference in frequency of sexual activity^28^. Scarring from surgery and stomas is another factor known to affect sexual confidence in patients with HSCR^29,30^. A relatively high frequency of sexual dysfunction has been reported for HSCR patients, primarily women, which could contribute to a lower rate of childbirth^13,14,23^. Another reason for the lower rate of childbirth in HSCR patients could be a fear of passing on the disease to their offspring. However, in theory, this should affect both men and women similarly.

Other malformations were significantly more common in HSCR patients compared with the controls and varied greatly from polydactyly to genital malformations. However, when adjusted for other malformations, the difference in the rate of childbirth remained. An infertility diagnosis did not differ between the HSCR patients and controls, despite a clear difference in the rate of childbirth for the female groups. The prevalence of an infertility diagnosis was very low for both groups. One plausible explanation for the lack of difference is the relatively young mean age in this study, as infertility is usually diagnosed later in life.

The Swedish registers used in this study have a high coverage and accuracy, which is one of the main strengths of this study. This made it possible to include a large study group, despite HSCR being a rare condition. The registers do not include details about surgical procedures, and therefore the impact of different surgical approaches on fertility was not assessed. The study design limits the authors to only examine the difference in fertility between HSCR patients and controls; it does not allow them to determine the cause. It can therefore only be speculated whether the difference in fertility is caused by mechanical damage due to inflammation or surgery, congenital reproductive anomalies, or voluntary childlessness, potentially affected by psychosocial factors. It was not possible to assess patients with assisted fertility in this study. A diagnosis of incontinence was used as a proxy for psychosocial factors. One of the limitations of using incontinence as a proxy is that some individuals might have been diagnosed with incontinence either during childhood, which may have resolved, or after childbirth, thus not having an effect on sexual activity and rate of childbirth. Another limitation is the young mean age of the groups. In Sweden, the mean age when the first child is born is 28–31 years, which fits well with the data for the current cohort^31^. Register studies do not enable an investigation of all aspects of the rather complex process of fertility. The rate of childbirth is not synonymous with fertility, but is a close proxy. As the rate of childbirth was collected from registers, it is not possible to verify whether there was a difference between the two groups regarding the desire to have children. The limitation of this register-based study was that it can only demonstrate an association and not the underlying mechanism of impaired fertility.

Further, adequately powered and controlled studies are needed to determine the cause of the impaired fertility in females with HSCR. However, the results highlight the importance of long-term follow-up of HSCR patients, especially the transition to adult care with appropriate access to gynaecological and fertility services.

## Supplementary Material

zrad043_Supplementary_Data

## Data Availability

Data can be accessed through Swedish National Registers.
